# Case Report: PET-avid compensatory tensor fascia lata hypertrophy mimicking metastatic disease in a patient with a chronic Achilles tendon rupture

**DOI:** 10.3389/fnume.2025.1699313

**Published:** 2026-01-12

**Authors:** Ian Strohbehn, James Graham, Christopher Kanner

**Affiliations:** 1 The Robert Larner MD College of Medicine, University of Vermont, Burlington, VT, United States; 2 Department of Radiology, University of Vermont Medical Center, Burlington, VT, United States

**Keywords:** biomechanics, compensatory muscle hypertrophy, FDG PET/CT, pseudomass, tensor fascia lata

## Abstract

In this case report, we describe an unusual case of PET-avid muscle enlargement in the right tensor fascia lata (TFL) muscle mimicking metastatic disease during lung cancer staging. The patient, a 67-year-old woman, had a history of left Achilles tendon rupture, which had been managed non-operatively. A PET/CT scan revealed focal fluorodeoxyglucose (FDG) uptake in a markedly expanded right TFL muscle. The presence of concurrent ipsilateral pelvic lymphadenopathy on the scan initially raised concern for muscle metastasis in this context. However, subsequent MRI of the hip showed diffuse muscle enlargement without a discrete mass, favoring benign muscle hypertrophy, particularly given the presence of a concurrent chronic Achilles tendon rupture that altered the patient's gait. A follow-up surveillance PET/CT and subsequent CT scans obtained over several years showed stability of the muscle abnormality, supporting the diagnosis of benign muscle hypertrophy and preventing an unnecessary biopsy. This case highlights the importance of correlating imaging findings with prior biomechanical injuries that can impact muscle architecture, particularly in a malignancy workup where FDG uptake in muscle may be misleading.

## Introduction

PET/CT is a well-established imaging technique in the diagnosis, staging, and monitoring of malignancy. Neoplastic cells often demonstrate increased glycolytic activity, resulting in higher FDG accumulation, which makes fluorodeoxyglucose (FDG)-PET/CT especially useful for the detection and staging of malignancy (1). However, there are myriad non-neoplastic causes of increased metabolic activity in tissues such as inflammation and infection that can mimic neoplasms on PET/CT and lead to false positives (2). We present a patient with newly diagnosed lung cancer whose staging PET/CT demonstrated focal uptake in an asymmetrically enlarged right tensor fascia lata (TFL) muscle. This case highlights a rare benign false positive cause of FDG activity in muscle, the need for contextual clinical correlation, and the diagnostic value of multimodality imaging.

## Case description

**Figure 1:**
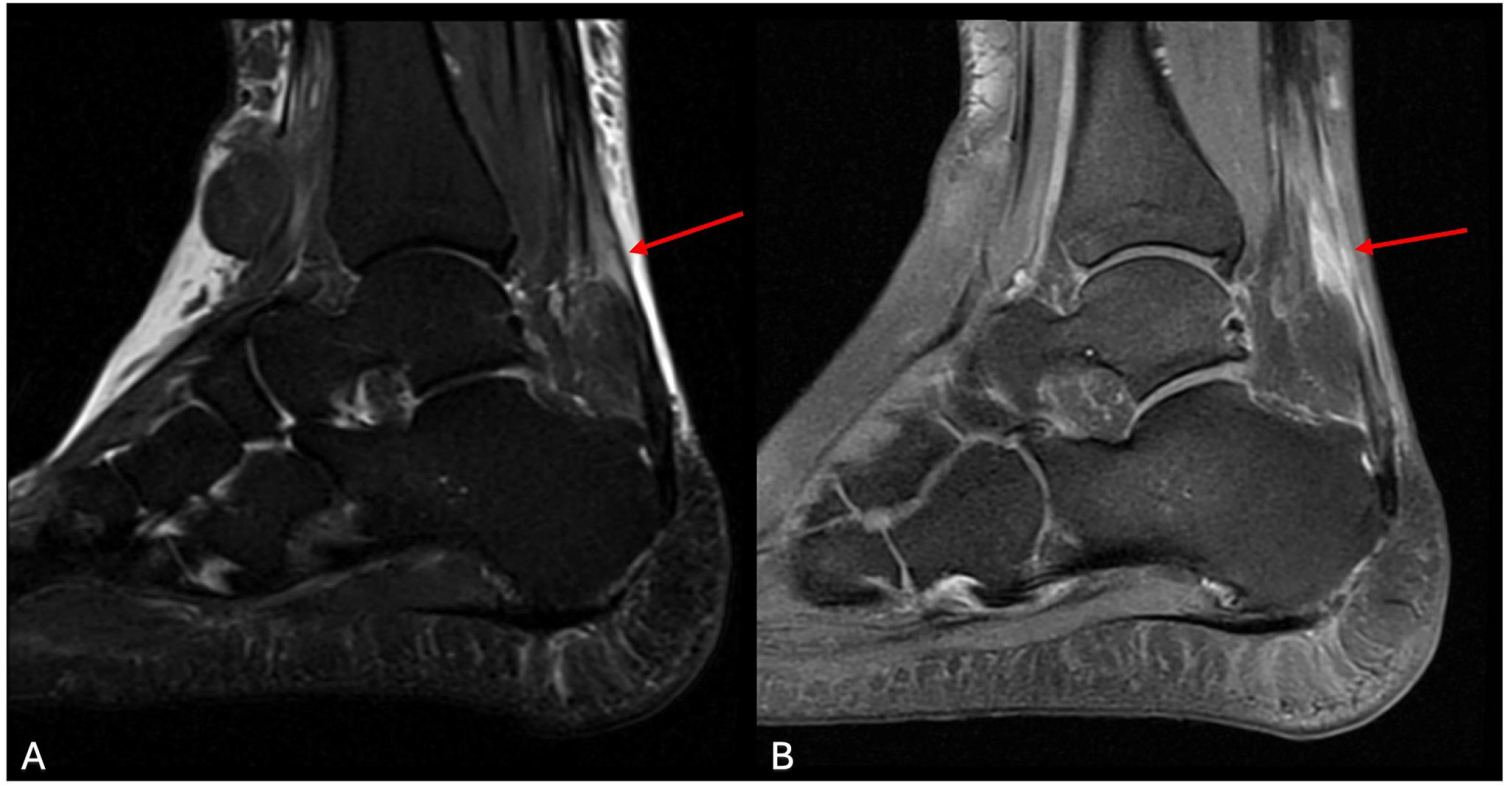
**(A)** T2 sagittal and **(B)** T1 post-contrast MRI show chronic disruption of the left Achilles tendon (red arrows).

This patient was a 67-year-old woman with a history of myocardial infarction, coronary artery disease, hypertension, hyperlipidemia, gastroesophageal reflux disease, obstructive sleep apnea, hypothyroidism, psoriasis, obesity, and non-alcoholic steatohepatitis. She presented to primary care in 2017 with 2 weeks of left posterior ankle pain and swelling after dancing. Treated initially with a corticosteroid injection for presumed Achilles tendinitis, she was later referred to physical therapy, where persistent pain and decreased dorsiflexion raised suspicion of tendon rupture. Ultrasound and MRI confirmed a complete left Achilles tendon rupture (Figure 1). Due to psoriatic arthritis and concerns over impaired wound healing, orthopedic surgery recommended conservative management, and she achieved functional recovery without operative intervention.

**Figure 2:**
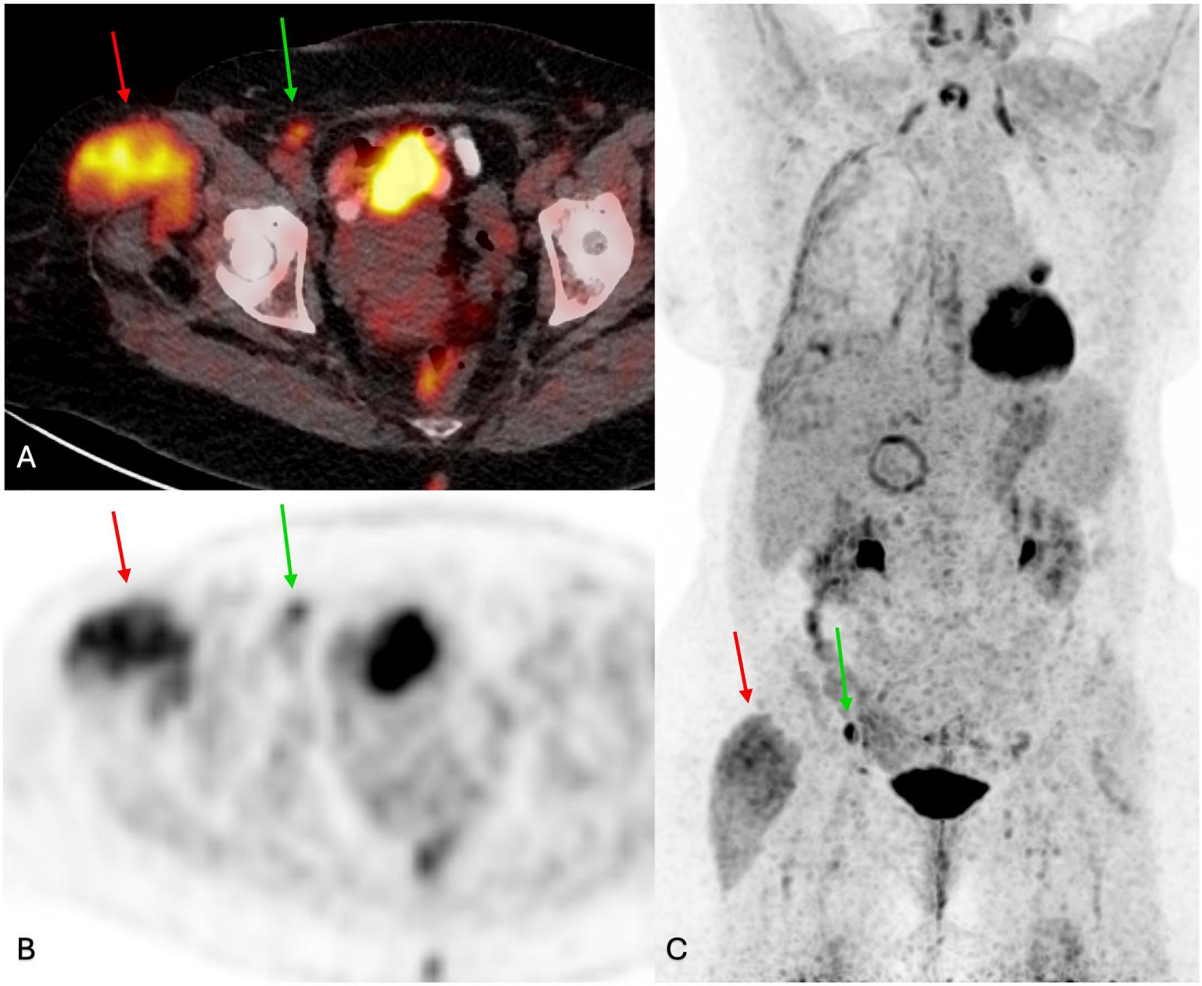
**(A)** Fused FDG PET/CT, **(B)** axial PET, and **(C)** PET MIP images show hypertrophied, FDG-avid, right tensor fascia lata muscle (red arrow, SUV max 5.96, background hepatic SUV max 2.86). FDG-avid inguinal lymphadenopathy is also present (green arrow, SUV max 3.58).

**Figure 3:**
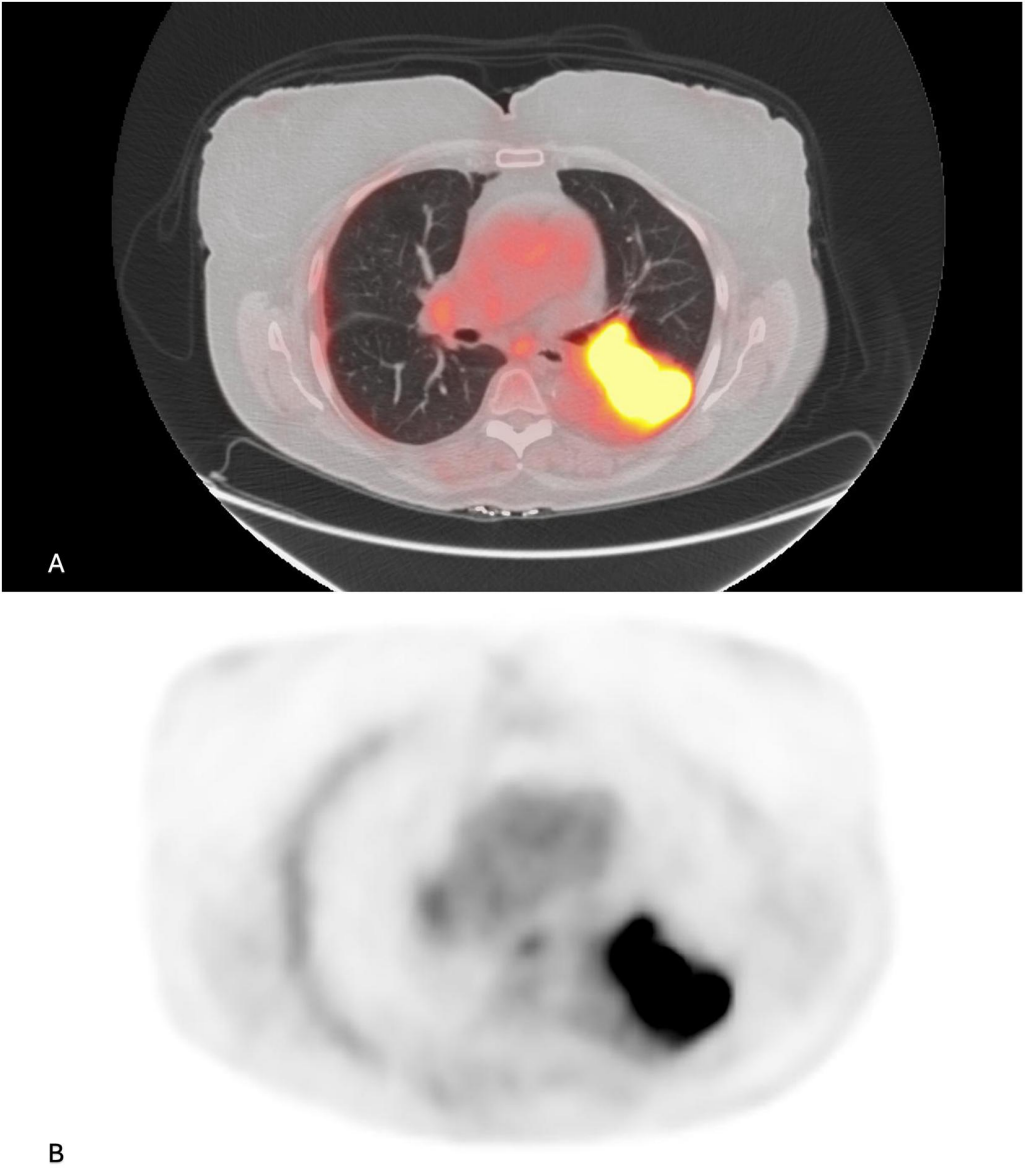
**(A)** Fused FDG PET/CT and **(B)** PET images show a lobulated, FDG-avid, left lower lobe lung mass (SUV max: 24.89).

Almost 4 years later, in 2021, the patient was evaluated for shortness of breath and a chest CT scan revealed a left lung mass with pleural effusions suggestive of malignancy. PET/CT performed 1 month later showed hypermetabolic activity in the lung lesion consistent with malignancy. However, it also incidentally demonstrated circumferential enlargement of the right TFL muscle, measuring 5.0 cm × 8.2 cm, with mild FDG uptake, asymmetrical fatty atrophy of the gluteal muscles, and ipsilateral FDG-avid inguinal and external iliac lymphadenopathy (Figure 2). A biopsy of the left lung mass ultimately confirmed a diagnosis of Stage IIIA non-small cell lung cancer, per the TNM Classification of Malignant Tumors, 8th edition (Figure 3). The etiology of the increased FDG uptake in the TFL was unclear, and the patient reported no pain or other symptoms in the right hip. A contrast-enhanced MRI scan of the right hip was recommended to further evaluate the PET/CT findings in the right TFL muscle.

**Figure 4:**
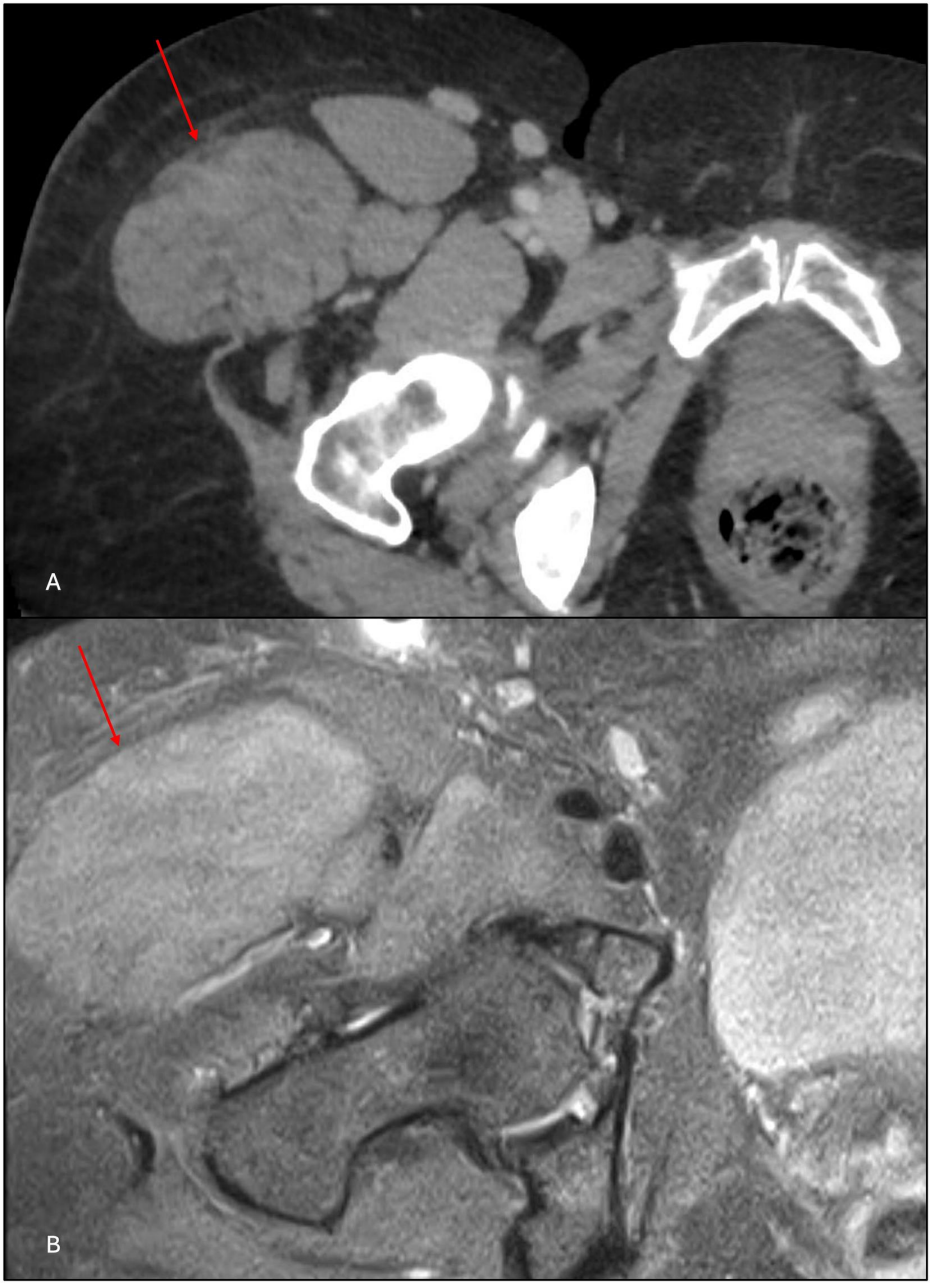
**(A)** Contrast-enhanced CT from January 2025 and **(B)** T1 fat-saturated MR images from October 2021 show right inguinal lymphadenopathy and enlargement of the right tensor fascia lata that are stable over 4 years.

The MRI scan demonstrated diffuse right TFL enlargement with preserved muscle architecture and no discrete enhancing mass (Figure 4). The findings were reviewed in a multidisciplinary sarcoma tumor board, and in the context of the patient's chronic altered gait from the left Achilles rupture, the consensus favored compensatory hypertrophy of the TFL rather than a neoplastic process. A follow-up PET/CT scan in 2022 demonstrated unchanged right TFL appearance and FDG uptake, and additional surveillance CT scans of the abdomen and pelvis performed as recently as 2025 showed long-term stability of this muscle abnormality (Figure 4). Based on imaging stability, the absence of a discrete mass on MRI, and the patient’s corroborative clinical history supporting functional muscle hypertrophy, biopsy was deemed unnecessary.

## Discussion

While rare, skeletal muscle metastasis from a primary lung malignancy has been reported and may present as an incidental finding on routine surveillance imaging or as a painful soft tissue mass (3, 4). Therefore, unilateral muscle enlargement with increased FDG uptake on PET/CT must be regarded with a degree of suspicion, particularly in the case described herein, where there was also ipsilateral FDG-avid lymphadenopathy. Primary muscular neoplasm must also be considered, given the diffuse nature of enlargement and high glycolytic metabolism present on FDG PET/CT. However, non-neoplastic processes, such as inflammatory myopathy, denervation changes, infection, and, in this case, compensatory muscle hypertrophy, can present similarly on PET/CT (5, 6) and should be considered plausible and, in fact, more likely alternatives. In addition, this report underscores a relatively common diagnostic pitfall in the interpretation of FDG PET/CT in oncologic imaging, namely a false positive exam from benign musculoskeletal processes (2, 6). Through correlation with clinical history and supplemental imaging, this observation could ultimately be attributed to compensatory hypertrophy due to chronic contralateral Achilles tendon rupture causing altered gait mechanics.

In this case specifically, in the setting of a neoplastic lung mass, PET-avid muscle enlargement in the right TFL raised concern for metastasis or primary soft tissue neoplasm. The presence of ipsilateral FDG-avid inguinal lymphadenopathy further elevated the concern for disseminated disease. However, further evaluation with MRI revealed normal, albeit hypertrophied muscle and no discrete intramuscular mass. The imaging findings were stable over time (confirmed by repeat PET/CT more than a year later and CT of the patient’s abdomen and pelvis up to 4 years later), and clinical correlation with the patient's prior left Achilles tendon rupture and lack of pain or other subjective abnormality in the right hip was pivotal in establishing the most likely etiology. This case showcases a rare instance of TFL hypertrophy mimicking the appearance of malignancy on PET/CT in a patient undergoing staging for a presumed primary lung malignancy. The absence of a tissue biopsy to confirm benignity could be perceived as a potential limitation in this case; however, the longstanding imaging stability of the pseudomass was considered adequate to exclude more ominous pathology, and the ability to forego a biopsy in this context is a major strength of the workup.

Compensatory muscle hypertrophy is a known biomechanical adaptation seen in response to increased mechanical load, typically due to contralateral limb weakness, deformity, or paralysis. There have been case reports of unilateral TFL hypertrophy and development of a pseudomass in response to biomechanical alterations, such as underlying abductor tendon tears, lumbar radiculopathy, and other biomechanical disturbances (7–11). Although such adaptations are well recognized, their appearance on PET/CT and potential to mimic malignancy rarely arises in practice. To our knowledge, this is the first reported case in which PET/CT demonstrated increased FDG activity in a TFL pseudomass, reflecting increased metabolic activity from overuse due to a contralateral chronic Achilles tendon rupture.

## Conclusion

Compensatory muscle hypertrophy can present as unilateral FDG-avid muscle enlargement (i.e., pseudomass) on PET/CT, which can mimic malignant disease, particularly in patients undergoing staging for a known malignancy. In this case report, we have described an unusual example of this potential PET pitfall, along with a suitable process to avoid an unnecessary muscle biopsy using clinical correlation, supplemental imaging, and multidisciplinary tumor board consensus. Skeletal muscle metastasis is rare, particularly in primary lung malignancy, and with an appropriately vigilant evaluation of the clinical context, it can be excluded by attributing a muscle pseudomass to a plausible mechanism, in this case, compensatory hypertrophy of the tensor fascia lata due to chronically altered gait from a contralateral lower extremity injury.

## Data Availability

The original contributions presented in the study are included in the article/Supplementary Material, further inquiries can be directed to the corresponding author.
